# Prevalence of intestinal schistosomiasis in pre-school aged children: a pilot survey in Marolambo District, Madagascar

**DOI:** 10.1186/s40249-021-00871-y

**Published:** 2021-06-25

**Authors:** Caitlin Sheehy, Heather Lawson, Emmanuel H. Andriamasy, Hannah J. Russell, Alice Reid, Gina U. Raderalazasoa, Graham Dodge, Robbie Kornitschky, James M. StJ. Penney, Tahiry N. Ranaivoson, Antsa Andrianiaina, Jenny S. Emmanoela, Amaya L. Bustinduy, J. Russell Stothard, Louis Andrianjaka, Stephen A. Spencer

**Affiliations:** 1 The University of Manchester Medical School, University of Manchester, Manchester, M13 9NT UK; 2 NHS Greater Glasgow and Clyde, Glasgow, G4 0SF UK; 3 Faculté de Médecine, Université D’Antananarivo, Antananarivo, Madagascar; 4 Department of Tropical Disease Biology, Liverpool School of Tropical Medicine, Liverpool, L3 5QA UK; 5 Department of Imaging, Brighton and Sussex University Hospital NHS Trust, Brighton, BN2 5BA UK; 6 Department of Clinical Research, London School of Hygiene & Tropical Medicine, London, UK; 7 Ministère de La Santé Publique, Antananarivo, Madagascar; 8 Postgraduate Medical Centre, Royal United Hospitals Bath NHS Foundation Trust, Bath, BA1 3NG UK

**Keywords:** *Schistosoma mansoni*, Soil-transmitted helminthiasis, Paediatric, Praziquantel, Mass drug administration

## Abstract

School-aged children (SAC) have a considerable burden of intestinal schistosomiasis in Madagascar yet its burden in pre-school aged children (PSAC) is currently overlooked. To assess the at-risk status of PSAC, we undertook a pilot epidemiological survey in June 2019 examining children (*n* = 89), aged 2–4-years of balanced gender, in six remote villages in Marolambo District, Madagascar. Diagnosis included use of urine-circulating cathodic antigen (CCA) dipsticks and coproscopy of stool with duplicate Kato-Katz (K-K) thick smears. Prevalence of intestinal schistosomiasis by urine-CCA was 67.4% (95% confidence interval [*CI*]: 56.5–77.2%) and 35.0% (95% *CI*: 24.7–46.5%) by K-K. The relationship between faecal eggs per gram (epg) and urine-CCA G-scores (G1 to G10) was assessed by linear regression modelling, finding for every increment in G-score, epg increased by 20.4 (6.50–34.4, *P* = 0.006). Observed proportions of faecal epg intensities were light (78.6%), moderate (17.9%) and heavy (3.6%). Soil-transmitted helminthiasis was noted, prevalence of ascariasis was 18.8% and trichuriasis was 33.8% (hookworm was not reported). Co-infection of intestinal schistosomiasis and soil-transmitted helminthiasis occurred in 36.3% of PSAC. These results provide solid evidence highlighting the overlooked burden of intestinal schistosomiasis in PSAC, and they also offer technical  guidance for better surveillance data for the Madagascan national control programme.

**Supplementary Information:**

The online version contains supplementary material available at 10.1186/s40249-021-00871-y.

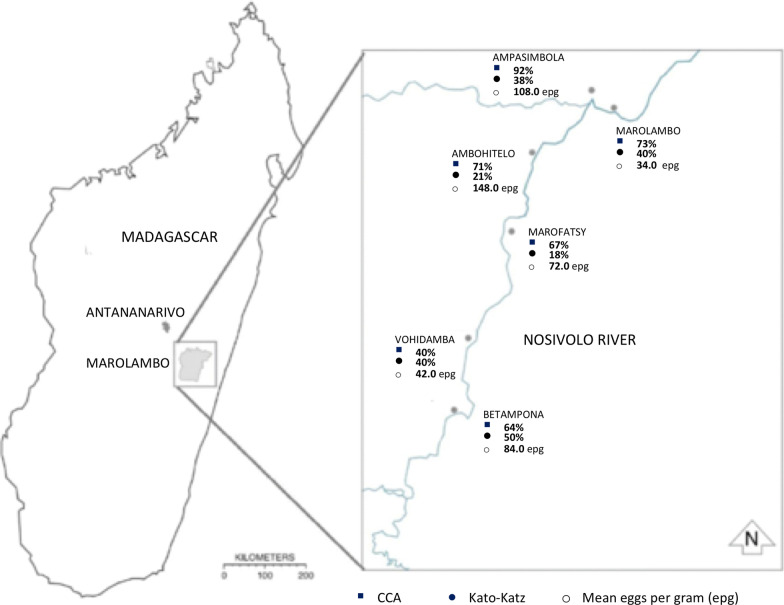

## Background

Despite substantial control efforts, schistosomiasis remains to be a significant global public health problem, particularly in sub-Saharan Africa and Madagascar [1]. In Madagascar, 107/114 districts are endemic for schistosomiasis, with the national control programme offering praziquantel to school-aged children (SAC), aged 5-15,  only through school-based mass drug administration (MDA) [2]. Given the remoteness of some disease endemic villages, many logistical challenges exist and perpetuate high rates of (re)infection. Recent epidemiological studies of SAC have documented very high burdens of infection and disease [3]. Both *Schistosoma haematobium* and *Schistosoma mansoni* exist on Madagascar; *S. haematobium* causes urogenital schistosomiasis and is present in northern and western districts; *S. mansoni,* which causes intestinal schistosomiasis, is present in eastern and southern districts; there are areas of co-endemicity in four regions in north-central and south-western parts of the country [2]. * Schistosoma mansoni* infection typically presents with bloody diarrhoea, abdominal pain and bloating [4]. With application of portable ultrasonography, Malagasy children as young as six have detectable periportal liver fibrosis, inferring that even younger children have developed chronic intestinal schistosomiasis [5].

Across mainland Africa, an estimated 50 million African pre-school aged children (PSAC) live with schistosomiasis [6], but PSAC are not yet formally included in national control strategies [7–9]. Feasible treatment options for PSAC include praziquantel crushed tablets administered on a test-and-treat basis or can be delivered in the WHO preventive strategy of ‘Integrated Management of Childhood Illnesses’ (IMCI) clinics [10, 11]. In contrast to Africa, there are no published reports of schistosomiasis among PSAC on Madagascar meaning their plight is currently overlooked. To address this and better inform the national intervention strategy, our pilot study builds on prior findings from SAC by MADEX research expeditions [3], to assess the current prevalence and infection intensity of intestinal schistosomiasis and soil-transmitted helminthiasis (STH) among PSAC in Marolambo District, Madagascar.

## Methods

### Study site and population

**Fig. 1:**
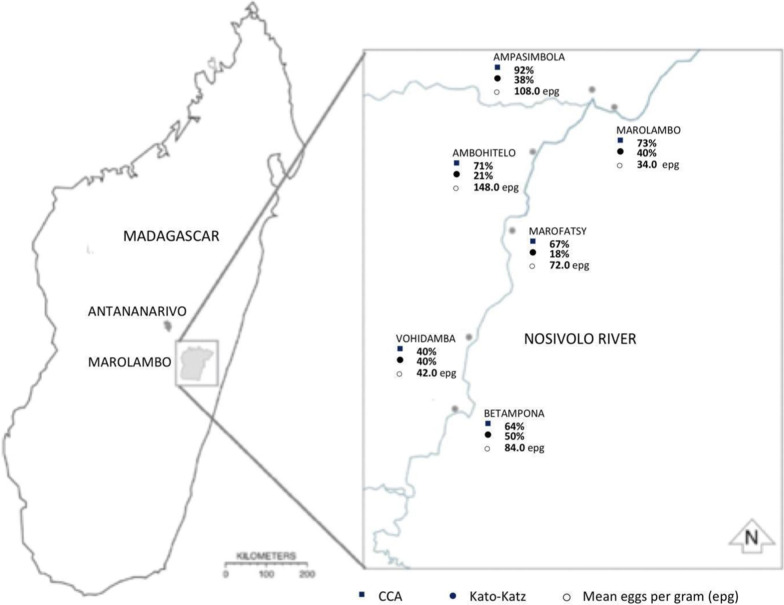
Map of Marolambo District, Madagascar showing the prevalence of *Schistosoma mansoni* among 2–4 year-old pre-school children in each village. Prevalence by both CCA and Kato-Katz are shown in addition to the arithmetic mean epg of positive PSAC in each village. *CCA* Circulating cathodic antigen, *PSAC* Pre-school-aged children

A cross-sectional survey was conducted between June-July 2019 in the hard-to-reach Marolambo District, eastern Madagascar. The population is estimated to be 250 000 people, of whom 95% are farmers. The primary source of water is the Nosivolo River (Fig. 1). We recruited 89 children aged 2–4-years from six villages: Marolambo, Ampasimbola, Ambohitelo, Marofatsy, Vohidamba, Betampona; the same sites of previous MADEX studies on *S. mansoni* associated morbidity [3, 5]. In each village 14–15 PSAC, aged 2–4-years old, were recruited through convenience sampling. Following community engagement announcing research intentions, parents with PSAC were asked if they would be willing to volunteer their children for the investigation. Owing to logistical constraints of available diagnostic urine-CCA dipsticks, only the first 15 children in each village were examined.

### Parasitology

Children were provided with individual sterile sample pots labelled with their unique identifiable number (IN) which they returned the following day containing their mid-stream urine samples and stool samples. Urine was not assessed for *Schistosoma haematobium* as this species is not endemic in Marolambo District [3]. Urine samples were tested for circulating cathodic antigen (CCA; Rapid Medical Diagnostics Tests, Pretoria, South Africa) and recorded by the G-scores (G1 to G10) system (Fig. 3)**;** a G-score of 1 was considered negative, all other scores were considered positive and ranked in ascending order with increasing visual intensity of the test band [12]. Urine CCA is a rapid, point-of-care test for *S. mansoni*, with a sensitivity of 83% and a specificity of between 81 and 90%, and when used in conjunction with coproscopy gains in specificity [13, 14]. Faecal coproscopy was undertaken by inspection of duplicate thick Kato-Katz (K-K) smears (Vestergaard-Frandsen, Lausanne, Switzerland) for *S. mansoni* and for soil-transmitted helminthiasis. According to World Health Organization (WHO) guidelines, *S. mansoni* infection was classified by intensity as light (1–99 eggs per gram; epg), moderate (100–399 epg), or heavy (≥ 400 epg); *Ascaris lumbricoides* infection intensity was classified as light (1–4999 epg), moderate (5000–49 999 epg) and heavy (≥ 50 000 epg); and *Trichuris trichiura* infection intensity was classified as light (1–999 epg), moderate (1000–9999 epg) and heavy (≥ 10 000 epg) [15]. The presence of hookworms was not recorded for it was not possible to read all K-K slides within the required one-hour window from slide preparation. As a diagnostic quality control, 10% of K-K slides were cross-checked by The Helminthiases Unit of the Institut Pasteur de Madagascar (IPM).

All *S. mansoni* infected children were treated with crushed praziquantel at 40 mg/kg under direct supervision. STH infected children were treated with a single dose of albendazole. Parents were requested to report back to the survey team should the child experience any immediate discomfort.

### Statistical analysis

All results were recorded on electronic tablets in the field. Statistical analyses were performed on Stata 2017 (Stata Statistical Software 15. College Station, TX: StataCorp LLC). Chi-squared and Fisher’s exact tests were used to assess for association between prevalence by both urine-CCA and K-K, with age, gender and village.

## Results

### *S. mansoni* prevalence and infection intensity

**Fig. 2:**
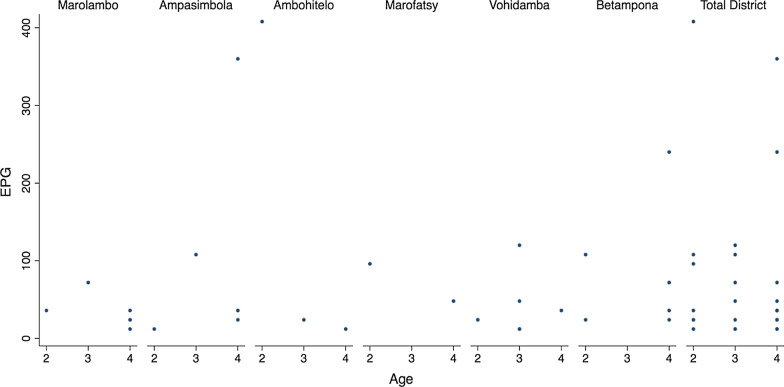
Scatter plot of epg data points of infected PSAC by age, for each village. Note the number of dots does not reflect the total number of individuals infected in each village (as detailed in Table 1) as some children had the same epg counts (with overlapping dots). This explains why there are more dots for four-year-olds than two- or three-year-olds. *PSAC* Pre-school-aged children, *EPG* Eggs per gram

**Fig. 3:**
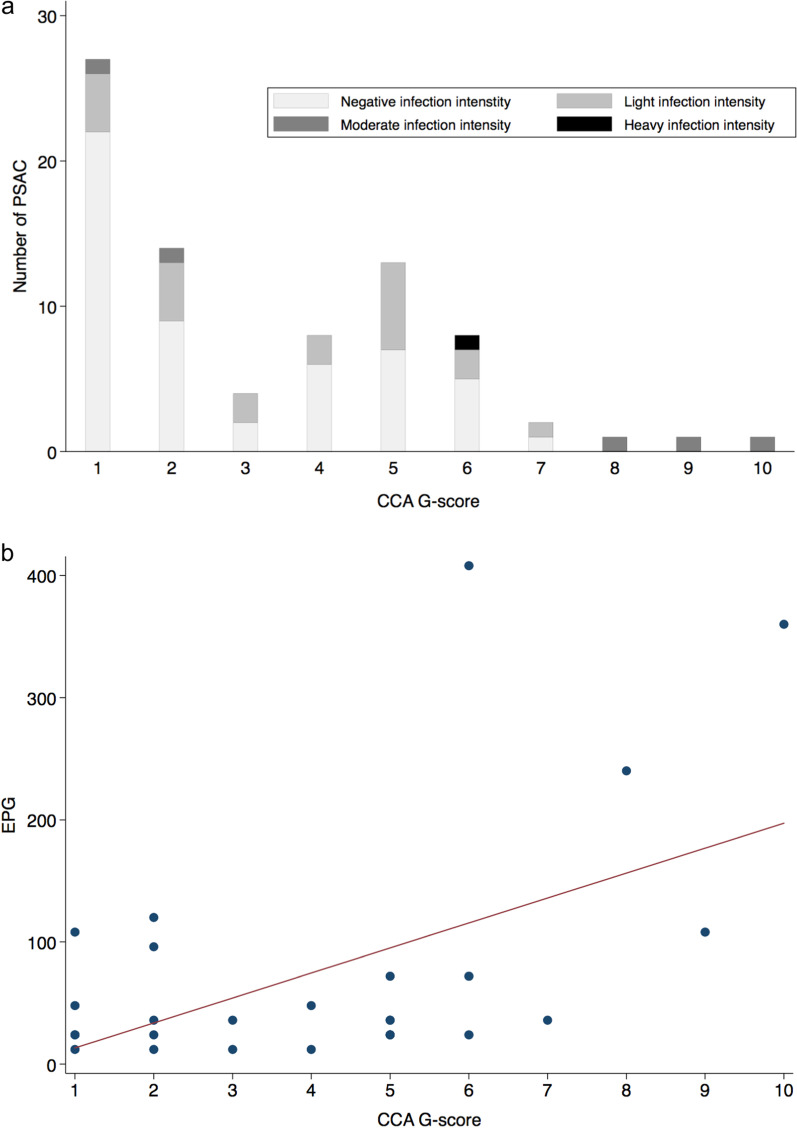
CCA G-score results: **a** number of PSAC with each G-score and **b** correlation between G-score and epg. **A** Frequency histogram of CCA G-scores shows the number of PSAC with each G-score. A child with a score of G1 was considered not-infected, and scores G2-10 were considered to be infected with *Schistosoma mansoni.* **B** Scatter plot of epg values against CCA G-score values, with linear regression line shown. The linear regression model estimated, that in this cohort, for every increase in G-score by a value of one, the epg increased by 20.4 (6.50–34.4, *P* = 0.006). *CCA* Circulating cathodic antigen, *PSAC* Pre-school-aged children

Our PSAC cohort consisted of balanced gender (45M:44F). The prevalence of *S. mansoni* infection was 58/86 (67.4%; 95% *CI*: 56.5–77.2%) by CCA and 28/80 (35.0%; 95% *CI*: 24.7–46.5%) by Kato-Katz (Table 1). There were no statistically significant differences in prevalence by age, gender or location, from either urine-CCA or by K-K (Table 1). The arithmetic mean infection intensity of positive individuals was 74.6 epg on K-K (95% *CI*: 35.7–113.4). Of the 28 K-K positive children, light infections were observed in 22/28 (78.6%), moderate intensity infections in 5/28 (17.9%) and heavy intensity infections in 1/28 (3.6%). The youngest age of infection for any parasite was 2-years, noting 2-year old children could have moderate intensity infections across all sampled villages (Fig. 2). CCA G-score results were skewed towards lower scores, where 17.4% had a G-score of G2 and 1.2% had a G-score of G10 (Fig. 3).

**Table 1:** Characteristics of study population with results from CCA tests and Kato-Katz microscopy

	No. participants	*Schistosoma mansoni* CCA results			*Schistosoma mansoni* Kato-Katz results								*Ascaris lumbricoides* Kato-Katz results						*Trichuris trichiura* Kato-Katz results					
		CCA +	% (95% *CI*)	*P*	K-K +	% (95% *CI*)	*P*	Light (%)	Moderate (%)	Heavy (%)	Mean epg	Any STH (%)	*Ascaris* K-K + (%)	*P*	Light (%)	(Moderate (%)	Heavy (%)	Mean epg	*Trichuris* K-K + (%)	*P*	Light (%)	(Moderate (%)	Heavy (%)	Mean epg
Overall prevalence	89	58/86	67.4 (56.5–77.2)		28/80	35.0 (24.7–46.5)		22/28 (78.6)	5/28 (17.9)	1/28 (3.6)	74.6	34/80 (42.5)	15/80 (18.8)		11/15 (73.3)	4/15 (26.7)	0/15 (0.0)	4202.4	27/80 (33.8)		27/27 (100.0)	0/27 (0.0)	0/27 (0.0)	126.7
Age, years																								
2	23	17/22	77.3 (54.6–92.2)		7/21	33.3 (14.6–57.0)		5/7 (71.4)	1/7 (14.3)	1/7 (14.3)	101.1	8/21 (38.1)	4/21 (19.1)		3/4 (75.0)	1/4 (25.0)	0/4 (0.0)	4026.0	7/21 (33.3)		7/7 (100.0)	0/7 (0.0)	0/7 (0.0)	78.9
3	38	24/37	64.8 (47.5–79.8)		7/34	20.6 (8.7–37.90		5/7 (71.4)	2/7 (28.6)	0/7 (0.0)	56.6	16/34 (47.1)	7/34 (20.6)		5/7 (71.4)	2/7 (28.6)	0/7 (0.0)	3524.6	12/34 (35.3)		12/12 (100.0)	0/12 (0.0)	0/12 (0.0)	116.0
4	28	17/27	63.0 (42.4–80.6)	0.52^b^	14/25	56.0 (34.9–75.6)	0.02^b^	12/14 (85.7)	2/14 (14.3)	0/14 (0.0)	70.3	10/25 (40.0)	4/25 (16.0)	0.78^b^	3/4 (75.0)	1/4 (25.0)	0/4 (0.0)	5565.0	8/25 (32.0)	0.94^b^	8/8 (100.0)	0/8 (0.0)	0/8 (0.0)	184.5
Gender																								
Female	44	29/43	67.4 (51.5–80.9)		17/42	40.5 (25.6–56.7)		14/17 (82.4)	2/17 (11.8)	1/17 (5.9)	62.8	18/42 (42.9)	10/42 (23.8)		9/10 (90.0)	1/10 (10.0)	0/10 (0.0)	3639.6	14/42 (33.3)		14/14 (100.0)	0/14 (0.0)	0/14 (0.0)	140.6
Male	45	29/43	67.4 (51.5–80.9)	1.0^a^	11/38	28.9 (15.4–45.9)	0.28^a^	8/11 (72.7)	3/11 (27.3)	0/11 (0.0)	92.7	16/38 (42.1)	5/38 (13.2)	0.23^a^	2/5 (40.0)	3/5 (60.0)	0/5 (0.0)	5328.0	13/38 (34.2)	0.93^a^	13/13 (100.0)	0/13 (0.0)	0/13(0.0)	111.7
Location																								
Marolambo	15	11/15	73.3 (44.9–92.2)		6/15	40.0 (16.3–67.7)		6/6 (100.0)	0/6 (0.0)	0/6 (0.0)	34.0	5/15 (33.3)	2/15 (13.3)		2/2 (100.0)	0/2 (0.0)	0/2 (0.0)	2454.0	4/14 (26.7)		4/4 (100.0)	0/4 (0.0)	0/4 (0.0)	81.0
Ampasimbola	15	12/13	92.3 (64.0–99.8)		5/13	38.4 (13.9–68.4)		3/5 (60.0)	2/5 (40.0)	0/5 (0.0)	108.0	9/13 (69.2)	2/13 (15.4)		2/2 (100.0)	0/2 (0.0)	0/2 (0.0)	1578.0	8/13 (61.5)		8/8 (100.0)	0/8 (0.0)	0/8 (0.0)	231.0
Ambohitelo	15	10/14	71.4 (41.9–91.6)		3/14	21.4 (4.7–50.8)		2/3 (66.7)	0/3 (0.0)	1/3 (33.3)	148.0	6/14 (42.9)	5/14 (35.7)		4/5 (80.0)	1/5 (20.0)	0/5 (0.0)	5260.8	5/14 (35.7)		5/5 (100.0)	0/5 (0.0)	0/5 (0.0)	105.6
Marofatsy	15	10/15	66.7 (38.4–88.2)		2/11	18.2 (2.3–51.8)		2/2 (100.0)	0/2 (0.0)	0/2 (0.0)	72.0	5/11 (45.5)	3/11 (27.3)		1/3 (33.3)	2/3 (66.7)	0/3 (0.0)	6600.0	3/11 (27.3)		3/3 (100.0)	0/3 (0.0)	0/3 (0.0)	44.0
Vohidamba	15	6/15	40.0 (16.3–67.7)		6/15	40.0 (16.3–67.7)		5/6 (83.3)	1/6 (16.7)	0/6 (0.0)	42.0	2/15 (13.3)	1/15 (6.7)		1/15 (6.67)	0/15 (0.0)	0/15 (0.0)	12.0	1/15 (6.7)		1/1 (100.0)	0/1 (0.0)	0/1 (0.0)	12.0
Betampona	14	9/14	64.3 (35.1–87.2)	0.10^b^	6/12	50.0 (21.1–78.9)	0.55^b^	4/6 (66.7)	2/6 (33.3)	0/6 (0.0)	84.0	7/12 (58.3)	2/12 (16.7)	0.84^b^	1/2 (50.0)	1/2 (50.0)	0/2 (0.0)	4428.0	6/12 (50.0)	0.54^b^	6/6 (100.0)	0/6 (0.0)	0/6 (0.0)	96.0

*CCA* Circulating cathodic antigen, *CI*  Confidence interval, *STH* Soil transmitted helminths

^a^Chi-Squared Test

^b^Fishers’s Exact Test

The linear regression model estimated that in our PSAC cohort, for every increase in G-score by a value of one, the epg increased by 20.4 (6.50–34.4, *P* = 0.006; Fig. 3). Of note, there were 4/27 (14.8%) and 1/27 (3.7%) of children with a G-score of G1 (as judged not infected) yet found to have light and moderate intensity infections by Kato-Katz, respectively (Fig. 3). Similarly, there were 30/52 (57.7%) children negative by Kato-Katz, who were found to have G-scores > 1 by CCA.

By combining the treatment naïve SAC epg results from 2015, with our treatment naïve PSAC epg results, an updated simple linear regression estimation predicts that the first age of infection is likely to be between 4–12 months (see Additional file 1: Figure S1). Although the estimates from this model assume that the epg of 2015 PSAC is the same as 2019 PSAC, for the purpose of estimating the first age of infection this is likely to be more accurate than incorporating epg data from SAC who have received PZQ due to the loss of the age-prevalence correlation [16].

### Soil-transmitted helminthiasis prevalence and infection intensity

The prevalence of STH infection was 34/80 (42.5%); ascariasis was identified in 15/50 (18.8%), trichuriasis among 27/80 (33.8%), and there was 8/80 (10%) with both infections. All ascariasis was of light infection intensity, while 11/15 (73.3%) individuals with trichuriasis had light intensity infections with the remainder 4/15 (26.7%) of moderate intensity. No heavy intensity infections of STHs were identified. Co-infection with *S. mansoni* and STHs existed in 29/80 (36.3%) of participants (by CCA for *S. mansoni* and Kato-Katz for STH) and was 12/80 (15.0%, by Kato-Katz for both *S. mansoni* and STH).

## Discussion

This pilot study is the first step in drawing attention to the burden of schistosomiasis amongst pre-school-aged (PSAC) in Madagascar. Despite a limited sample size of 15 children per village, we report on alarming levels of intestinal schistosomiasis in young children currently ignored. Upon consideration of egg-patent infections, 35.0% (95% *CI*: 24.7–46.5%) of 2–4-year-olds were found to have intestinal schistosomiasis. This is unsurprising given that previous studies have demonstrated the Marolambo District to be hyperendemic for intestinal schistosomiasis, with school-aged children (aged 5–14) having prevalence of 94% by CCA and 74% by Kato-Katz [3].

Given the insensitivity of Kato-Katz methodology in the detection of light or early acquired infections (where worm pairs might not have reached full fecundity), the higher recorded CCA prevalence of 67.4% (95% *CI*: 56.5–77.2%) is to be expected [17]. The majority of infected individuals had light egg-patent intensity infections (76.8%) and some with moderate (17.9%) and heavy intensity infections (3.6%). These prevalence and infection intensity results approximate to the statistical estimates extrapolated from treatment naïve SAC data from 2015 from the same six villages; a study found a positive correlation between age and both prevalence and infection intensity, predicting light to moderate infections in approximately 50% of 2–4-year-old pre-school children [3]. As heavy infections were found from 2-year-olds (in Ambohitelo village), this further points towards an earlier age of first exposure to schistosome cercariae, and is in keeping with observations from both Kenya and Côte d’iVoire where children as young as 5 and 6 months old (respectively) have been found to have *S. mansoni* infection [18, 19]. This is of importance when considering treatment age for future schistosomiasis control policies.

There are a number of limitations to consider when interpreting our findings. The convenience sampling may have resulted in selection bias. Specifically, those who volunteered may have been more engaged with schistosomiasis education and prevention activities and therefore skewed results towards lower disease burdens. Alternatively, parents with concerns about their child’s health may have been more willing to volunteer their children for participation in the study, skewing the results towards higher prevalence and infection intensity. This pilot study enrolled a small sample of 80 participants as it was the first step in assessing prevalence amongst PSAC. This small sample size is reflected by large confidence intervals around both prevalence estimates as well as for the association between G score and epg. A larger and more robust study design would likely yield more accurate and representative associations between epg and G scores in this population. Despite these limitations, this study provides important preliminary data about the prevalence of schistosomiasis in PSAC. There is a need for further studies into the optimal dosing, frequency and timing of paediatric praziquantel treatment as well as the need for efforts to develop other interventions that can bring broader benefit to these afflicted communities, not forgetting the fine-scale spatial heterogeneities of typical schistosomiasis here and elsewhere.

To our knowledge, this is the first study to present the measurement accuracy of CCA G-score on PSAC. A proportion of G1-scores (18.5%; as judged not infected) were reported in children with evidence of egg-patent infection. Studies among SAC have previously reported the CCA test specificity to be between 81 and 90%, where CCA tests have yielded negative results in egg-patent individuals with evidence of light infections, and less commonly with moderate or heavy infections [13, 14]. The reason for this remains unclear but may be due to physiological differences in urinary excretion of CCA between children.

Across sub-Saharan Africa there is increasing recognition of schistosome infection among PSAC [20, 21]. Untreated, chronic infection leads to significant and detrimental effects on health, including stunting, wasting, anaemia, reduced exercise tolerance, impaired cognitive development or even death from the consequences of periportal liver fibrosis [4, 10]. Calprotectin and faecal occult blood tests have shown to be effective biomarkers for morbidity in *S. mansoni* in children as young as 3-years who have been found to have associated anaemia and should be attempted to be used in this setting here to better describe the burden of disease in PSAC [22]. Nonetheless, evident progression towards liver disease is clear since our 2016 study identified 11% of SAC had sonographic evidence of liver fibrosis, from 6-years of age [5]. Similarly, research in Côte d’Ivoire has demonstrated sonographic evidence of bladder lesions in PSAC infected with *S. haematobium* that intensifies without treatment [23]. As PSAC in Madagascar are not yet included in the national control programme [24], many already suffer chronic disease which will only progress further if left untreated before primary school age. To prevent disease progression, access and delivery of praziquantel to PSAC is needed; waiting to provide treatment until children are over 5-years of age is not justifiable in the context of already evident chronic disease [11].

The anthelmintic praziquantel is well recognised to be both a safe and efficacious treatment among PSAC [6]. PSAC dosing is extrapolated from adult dosing regimens using 40 mg/kg praziquantel crushed in a syrup drink to reduce the bitter taste of praziquantel [25, 26], although pharmacokinetic and pharmacodynamic data suggests higher doses up to 60 mg/kg may be needed [27], and are currently being investigated [28]. Efforts towards developing a paediatric orally dispersible praziquantel tablet for children aged 3-months to 6-years are underway with ongoing Phase III clinical trials at the time of writing [29, 30]. All infected children in our age-group were treated after the study with appropriate dose of praziquantel and the recommended single dose of albendazole for STH treatment in line with current dosing recommendations and we observed that there were no reported adverse effects.

Although WHO guidance from 2010 recommended a case-by-case approach to treatment of PSAC with *S. mansoni* infection*,* realising the pervasive nature of infections, preventive chemotherapy approaches through IMCI strategy, or MDA with praziquantel to include PSAC are reasonable [10]. Inclusion of PSAC in the Madagascar national control programme, for example first with expanded surveillance then inclusion in treatment campaigns using crushed praziquantel, whilst waiting for deployment of the paediatric praziquantel formula; this is justified as a much-needed step towards reducing its public health burden in both SAC and PSAC by 2025 [31]. There were 36.3% of children co-infected with schistosomiasis and STH in our study, which suggests that mass treatment with both albendazole and PZQ may be more practical and beneficial than test-and-treat.

Alternative interventions are also desired such as improving access to safe water, sanitation and hygiene (WASH) facilities, health education for specific behavioural change as well as focal use of molluscicides [15]. In Marolambo, communities rely on the Nosivolo river as their main water source, for drinking, washing, cooking and transport; safer, alternative water sources are limited [3]. PSAC are at increased risk of infection and re-infection through passive water contact if caregivers regularly come into contact with infested water sources [21]. Strategies to combat this will have important impacts on transmission in children, particularly those less than 3-years of age [32]. In this setting, consideration of preventive chemotherapy for ascariasis and trichuriasis is also needed which could synergise future drug delivery campaigns to improve health of PSAC more broadly. In short, hyper-endemic regions such as this should be amongst the first to benefit from deployment of the forthcoming paediatric praziquantel formulation.

## Conclusions

This pilot study assesses the prevalence and infection intensity of S. *mansoni* and soil transmitted helminths amongst PSAC in a hard-to-reach region of Madagascar. It provides an important starting point to develop better evidence-based policies for control of schistosomiasis in Malagasy PSAC. We describe a significant burden of intestinal schistosomiasis which further informs policymakers of the pressing need to expand future access to praziquantel alongside developing strategies that synergise with other child health programmes.

## Supplementary Information

**Additional file 1: Figure S1.** Scatter plot and linear regression line (with 95% CI represented by the shaded area) of faecal eggs per gram (epg) by age. The data points included in this figure are from coproscopy by Kato-Katz from stool among treatment naïve (pre-MDA) school aged children (SAC) from June 2015 (3), and treatment naïve PSAC in June 2019. The simple linear regression line (epg = -14.9 + 48.1*Age), estimates that for each increase in age by one year, the epg increases by 48.05 (95% CI: 23.0–73.1).

## Data Availability

The datasets used and/or analysed during the current study are available from the corresponding author on reasonable request.

## Abbreviations

epg: Eggs per gram
CCA: Circulating cathodic antigen
*CI*: Confidence interval
IN: Identifiable number
IMCI: Integrated Management of Childhood Illness
K-K: Kato-Katz
MADEX: Madagascar medical expeditions
MDA: Mass drug administration
PC: Preventive chemotherapy
PSAC: Pre-school-aged children
SAC: School-aged children
STH: Soil-transmitted helminthiasis
WASH: Water, sanitation and hygiene
WHO: World Health Organization
