# Supplementary figures and images for: Prevalence of intestinal schistosomiasis in pre-school aged children: a pilot survey in Marolambo District, Madagascar
Source: Infect Dis Poverty. 2021 Jun 25;10:87. doi: 10.1186/s40249-021-00871-y (PMC8235251; doi:10.1186/s40249-021-00871-y)

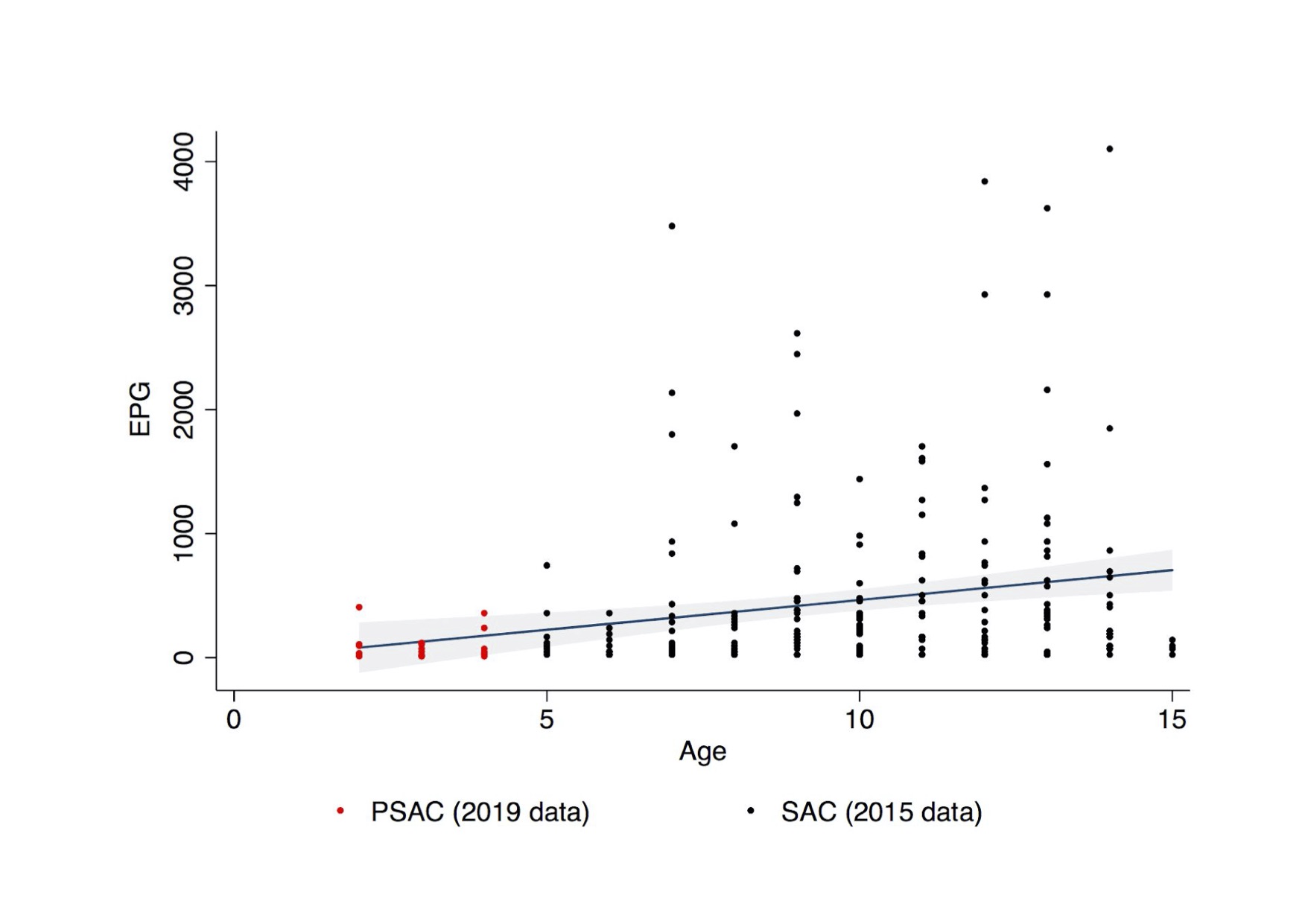

Supplement: Supplementary file 1 — Additional file 1: Figure S1. Scatter plot and linear regression line (with 95% CI represented by the shaded area) of faecal eggs per gram (epg) by age. The data points included in this figure are from coproscopy by Kato-Katz from stool among treatment naïve (pre-MDA) school aged children (SAC) from June 2015 (3), and treatment naïve PSAC in June 2019. The simple linear regression line (epg = -14.9 + 48.1*Age), estimates that for each increase in age by one year, the epg increases by 48.05 (95% CI: 23.0–73.1). [file 40249_2021_871_MOESM1_ESM.jpg]
